# Is it really a neuromyth? A meta-analysis of the learning styles matching hypothesis

**DOI:** 10.3389/fpsyg.2024.1428732

**Published:** 2024-07-10

**Authors:** Virginia Clinton-Lisell, Christine Litzinger

**Affiliations:** Department of Education, Health, and Behavior Studies, University of North Dakota, Grand Forks, ND, United States

**Keywords:** learning styles, meta-analysis, modality, systematic review, crossover interaction

## Abstract

Learning styles have been a contentious topic in education for years. The purpose of this study was to conduct a meta-analysis of the effects of matching instruction to modality learning styles compared to unmatched instruction on learning outcomes. A systematic search of the research findings yielded 21 eligible studies with 101 effect sizes and 1,712 participants for the meta-analysis. Based on robust variance estimation, there was an overall benefit of matching instruction to learning styles, *g* = 0.31, SE = 0.12, 95% CI = [0.05, 0.57], *p* = 0.02. However, only 26% of learning outcome measures indicated matched instruction benefits for at least two styles, indicating a crossover interaction supportive of the matching hypothesis. In total, 12 studies without sufficient statistical details for the meta-analysis were also examined for an indication of a crossover effect; 25% of these studies had findings indicative of a crossover interaction. Given the time and financial expenses of implementation coupled with low study quality, the benefits of matching instruction to learning styles are interpreted as too small and too infrequent to warrant widespread adoption.

## Introduction

Learning styles have been the topic of ongoing debate in education. Teacher education textbooks often state matching instruction to students’ preferred style will optimize learning outcomes (i.e., the *matching hypothesis;* Cuevas, 2015; Wininger et al., 2019). In contrast, cognitive scientists have argued there is a lack of empirical evidence to support the claims of the matching hypothesis (Kirschner, 2017; Willingham, 2018). Because of the lack of known empirical evidence supporting the matching hypothesis, there is understandable concern that perpetuating the concept of learning styles could lead to wasting resources (namely, educator time and effort) to match instruction as well as stereotyping students into restrictive categories (Newton and Miah, 2017). However, a meta-analysis aggregating findings compiled from an exhaustive search for studies on matching instruction to learning styles has not been conducted. Such a meta-analysis could be very helpful in informing this ongoing debate between educational practitioners and researchers. The purpose of this study was to conduct a meta-analysis of learning outcomes comparing conditions in which instruction is matched to students’ preferred learning styles to when instruction is unmatched to students’ preferred learning styles.

## Literature review

It is not controversial that there are substantial individual differences in student learning—teacher education and cognitive science scholars agree on this concept. There is substantial empirical evidence that students’ academic performance and learning vary due to background knowledge, motivation, and study strategies (Fong et al., 2021; Smith et al., 2021), just to name a few examples. However, the concept in learning styles theories that is controversial is the *meshing* or *matching hypothesis* in which students learn better when their instruction matches their preferred learning style (Pashler et al., 2008; Cuevas, 2015; Lyle et al., 2023). A key aspect of the matching hypothesis is that there is a crossover interaction (Kirschner, 2017), also known as a qualitative interaction, in which a particular treatment (in the case of learning styles, a particular modality of instruction) is effective for at least one subgroup but a different treatment is effective for another subgroup (Qiu and Wang, 2019). Generally speaking, these crossover treatment interactions are rare (Petticrew et al., 2012; Preacher and Sterba, 2019), but important to determining optimal treatments for individuals (Olsen et al., 2019; Qiu and Wang, 2019).

There are numerous learning styles (Dunn, 1990) as well as cognitive styles in which the preferred order of processing information varies (Calcaterra et al., 2005; Fiorina et al., 2007). The most prevalent are preferred modalities for learning information (Dekker et al., 2012; Brown, 2023). Learners are generally categorized through self-reports of preferred modalities (An and Carr, 2017), such as the VAK typology (visual, auditory, and kinesthetic; Fallace, 2023a,b). An example of accommodating these styles would be to provide information for learners categorized as “visual” in pictures, learners categorized as “auditory” would process the same information best aurally, and learners categorized as kinesthetic would have a hands-on activity (Dunn and Dunn, 1975). Then, a read/write category was added making it the VARK typology for learners who were thought to best process information through reading verbal information (as opposed to visual learners who better processed pictures; Fleming and Mills, 1992).

A typology similar to the VARK for categorizing learning styles is the verbalizer/visualizer approach (Riding and Rayner, 1998). According to this framework, verbalizers tend to mentally represent information in words whereas visualizers (also called imagers) tend to mentally represent information in mental pictures or diagrams (Riding and Sadler-Smith, 1992; Knoll et al., 2017). Subsequently, the developers of this framework argue that verbalizers better learn the material presented in text and images better learn the material presented in images (Riding and Sadler-Smith, 1992). This is analogous to the visual and read/write learners in the VARK model. Importantly, both the VARK and the verbalizer/visualizer approach advocate matching the modality of the instruction to the students’ learning style.

Adapting instruction based on modality learning styles may be conflated with multimodal instruction. Multimodal instruction is providing information to students in more than one modality, such as a text with relevant pictures or diagrams (Bouchey et al., 2021). The rationale for providing students with multiple modalities is grounded in dual coding in which visual and verbal information are processed in separate channels or pathways in the architecture of human cognition (Paivio, 1991; Reed, 2006). Having information presented in two modalities (and subsequently two channels) allows for more information to be processed at a given time (Mayer and Anderson, 1992; Mayer, 2011). Multimodal instruction has been found to benefit learning for students (Mayer, 2017; Noetel et al., 2022). However, it should be noted there are individual differences in the degree of benefit, such as students with lower levels of background knowledge tend to have more benefit from adding visuals to verbal information compared to their peers with higher levels of background knowledge (Mayer, 2017). This is distinct from learning styles in that certain students learn better than others in multimodal instruction, but there is not a crossover in which students receive harm or benefit from multimodal instruction. Individuals who support learning styles have been found to also support multimodal instruction (Nancekivell et al., 2021). However, matching instruction to learning styles is more time-consuming as it involves assessing for styles and purposefully assigning modalities, rather than providing multiple options available for all students.

There are concerns that matching instruction to learning styles relates to psychological essentialism, which is the belief that categories of people are innate and biologically based (Gelman, 2003; Nancekivell et al., 2020). An essentialist view of learning styles would be that, for example, visual learners are born with a predisposition to learning visually and that this limits what they can learn through other modalities. Indeed, essentialist and non-essentialist believers in learning styles have been identified (Nancekivell et al., 2020). Essentialist belief in learning styles may explain why visual learners are perceived as more intelligent and better performing academically than kinesthetic, “hands on,” learners (Sun et al., 2023). Relatedly, learners who are told they have a particular style may have a self-fulfilling prophecy in which they believe they can only learn in a particular modality and subsequently do not develop necessary skills in modalities outside of their style (Vasquez, 2009).

Given the resources involved and potential consequences relevant to psychological essentialism, learning styles would logically need to demonstrate remarkable efficacy to justify their use in education. In a review of learning styles efficacy, a team of cognitive scientists focused on student learning explained the criteria for validating the matching or meshing hypothesis (Pashler et al., 2008). One is to categorize learners based on a measure of learning style into at least two groups. A second is that participants need to be randomly assigned to receive instruction in a minimum of two methods (e.g., visual compared to auditory information). Learners need to be assessed in the same manner across styles and conditions. Finally, there needs to be a crossover in which there is an interaction between the learning style group and instruction in which matched instruction has higher learning gains than unmatched instruction for each of the learning style groups. This avoids the possibility that the instruction intended to be matched for a particular style is simply better across style groups. For example, college students who were prompted to visualize statements (visual matching) remembered more statements than their peers who were prompted to consider the sounds in the statements (auditory matching) across learning style categories (Cuevas and Dawson, 2018).

The review by Pashler et al. (2008) concluded that there was a lack of empirical evidence to support matching instruction to student’s learning styles that met their criteria for validating the matching hypothesis. Since this time, there have been other reviews similarly concluding that there is a lack of empirical support for matching instruction to students’ learning styles (Cuevas, 2015; Klitmøller, 2015; Aslaksen and Lorås, 2018). However, there has not been a meta-analysis aggregating effects across studies to provide an estimate of magnitude. Such an approach provides more precision that can be deduced from individual studies and more power to detect effects that may be provided by a single study sample (Deeks et al., 2023). Moreover, meta-analyses may help resolve controversies based on conflicting study findings (Deeks et al., 2023).

### Potential moderators

The modality of instruction for matching to learning styles should be considered when considering effects. For example, verbalizer or read/write learners may have their matched instruction involve reading and auditory learners would receive the same information aurally (e.g., Rogowsky et al., 2015, 2020; Lehmann and Seufert, 2020). However, reading comprehension is somewhat better than listening comprehension for inferential understanding in which readers need to connect ideas from the text (Clinton-Lisell, 2022). However, listening may be more effective than reading when accompanied by relevant visual representations, such as pictures or diagrams (Noetel et al., 2022). In addition, non-verbal images (pictures) tend to be remembered better than the same information presented in words (Paivio and Csapo, 1973).

The modality of the assessment should be considered as a potential moderator. Pashler et al.’s (2008) criteria understandably require the learning assessment to be the same modality in order to make comparisons between matched and unmatched instructions based on learning styles. However, this typically involves one method of instruction being in the same modality as the assessment and the comparison method of instruction being in a modality different than the assessment. For example, a listening task would be considered matched for auditory learners and a reading task would be considered matched for read/write or verbal learners and the assessment would be in writing, which is the same modality as the matched instruction for read/write or verbal learners. It is possible that there is a modality-match effect in which having the same modality at learning and assessment would affect results (Mulligan and Osborn, 2009). Encoding and producing information in the same modality may be easier than in different modalities (Staudigl and Hanslmayr, 2019), and subsequently, whether the instruction modality and assessment modality were the same should be considered.

Experimental studies comparing instructions matching and unmatched to learning styles have been conducted with between-subjects and within-subjects designs. With a between-subjects design, participants are in separate groups and only experience one condition. In the case of learning styles, participants would be placed in a group to either receive instruction matched or unmatched to their categorized learning style. An advantage to between-subject designs is that participants are unaware of conditions they were not assigned to thereby preventing carryover effects from other conditions as well as practice effects (Charness et al., 2012). However, different individuals are compared by condition, and subsequently, prior group differences could confound effects thought to be due to condition (Gray et al., 2003; Adesope et al., 2017). In contrast, a within-subjects design involves participants experiencing both instruction matched and instruction unmatched to their learning style with different materials and counterbalanced to prevent order effects. With a within-subjects design, each participant serves as their own control, which prevents prior group differences at baseline to confound results (Charness et al., 2012). Because these research designs are comparable, but not identical, it is recommended that the study design be tested as a moderator in meta-analyses (Borenstein et al., 2009).

Study quality is an important consideration in meta-analyses as it is possible for treatment effects to vary as a function of study quality (Sterne et al., 2001; Feeley, 2020). However, removing low-quality studies from analyses may lead to missing valuable data and meta-analyses should strive to be as inclusive as possible to have an accurate understanding of the accumulated evidence (Weaver, 2011; Feeley, 2020). Narrow inclusion criteria themselves may bias meta-analytic findings. Moreover, studies in social sciences and education (such as the ones for the current meta-analysis) tend to receive low-quality ratings due to methodological details (particularly internal consistency) not being reported (Singer and Alexander, 2017; Feeley, 2020). However, the potential influence of study quality should be considered by coding the quality of each study using predetermined quality criteria and including study quality as a moderator to assess its potential contribution to varying effects (Pigott and Polanin, 2020; see Austin et al., 2019; Zhu et al., 2021; Lam and Zhou, 2022).

### The current study

The purpose of this study was to conduct a meta-analysis of matching instruction to modality learning styles. In doing so, the criteria from Pashler et al. (2008) are generally followed. One exception is that non-randomized quasi-experiments are included given the valuable information they provide due to their external validity in education research (Waddington et al., 2022). Three research questions guide this inquiry:

1. What is the aggregated effect of matching instruction to learning styles compared to unmatched instruction on learning outcomes?
2. How frequent is the crossover of matching instruction by style? In other words, is there an interaction indicating benefits to matched instruction over unmatched instruction for at least two of the styles examined?
3. Does the study design (between or within subjects), type of styles, modality of instruction, or study quality moderate the effects of matching instruction to learning styles?

## Methods

The data extracted from the included studies and R code used for analyses are available on the Open Science Framework (Clinton-Lisell, 2023b).

### Author positionality

Following guidance from Castillo and Babb (2024), information about the authors’ backgrounds and identities is shared in this section.

The first author learned a cognitive approach to educational psychology during her doctoral and postdoctoral studies. During these times, she was taught that there was a lack of empirical evidence to support the concept of learning styles and that it was a common myth of education. Furthermore, she is aware that learning styles have origins rooted in ethnocentrism in which white scholars developed the concept based on condescending attitudes toward children of color (Fallace, 2019). As a white woman, this is a history she works to be mindful of not repeating.

The second author has a master’s degree in school counseling and was influenced by behavioral and school counseling theories. The career aspects of school counseling education supported the use of learning style inventories during the time she received her training. As a researcher, the second author became aware of learning styles research that did not support the career education practices being utilized in the educational setting. The second author shifted practices in her work away from using learning style inventories as part of career education because of the current research on the topic. As a white woman and first-generation college student, she works to be mindful of the social/cultural underpinnings that could influence the understanding of research in learning styles.

### Inclusion criteria

Following Pashler et al.’ (2008) guidelines, studies for the learning styles meta-analysis were included if they met the following criteria: (1) participants were categorized in at least two types of learning styles (e.g., visual and auditory), (2) there was at least one condition with instruction and/or learning materials matching to the participants’ learning styles and at least one condition with instruction and/or learning materials not matching to the participants’ learning styles, (3) the unmatched condition for one type of learning style was considered a matched condition for another learning style (so that a crossover interaction could be examined), (4) there was a measurement of learning that was identical across conditions and styles, (5) the study was disseminated in English because of the linguistic skills of the research team, and (6) descriptive statistics were reported to calculate effect sizes or the author of the study provided these upon request.

### Systematic search

**Figure 1:**
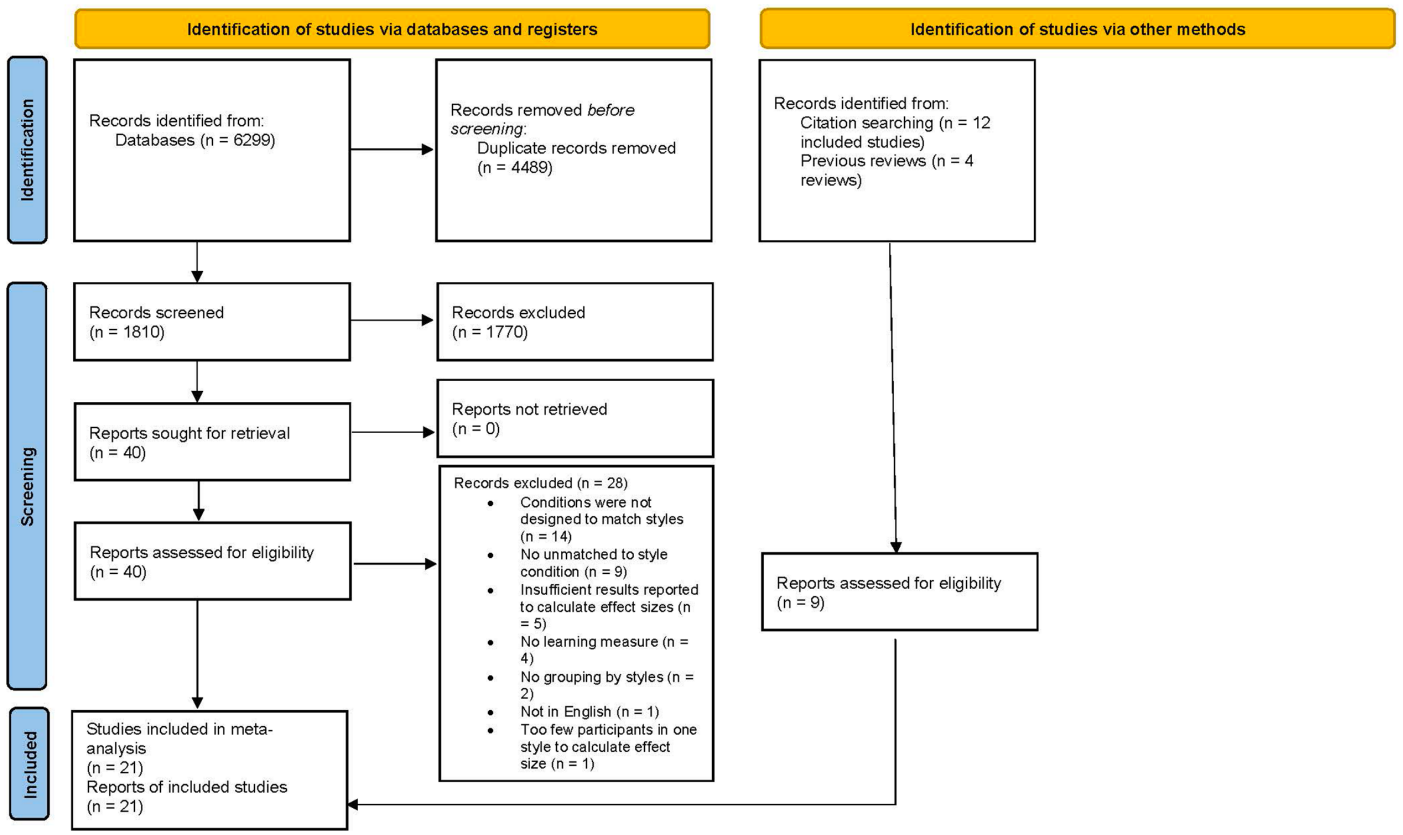
PRISMA flow chart of the systematic review process.

The first step in the systematic search for relevant articles included a broad search of the databases Web of Science, Scopus, PsycInfo/EBSCOhost, ERIC, and ProQuest Dissertations and Theses using the search terms such as “learning style*” and “learning preference*.” Dissertations and theses were important to include in the search as they are less likely to be influenced by publication bias in which journal articles are more likely to get published when reporting significant results (Paez, 2017). A total of 6,299 citations were found (see Figure 1 for a flow chart of the systematic search process). After duplicates were removed, 1,810 remained. These citations were screened based on titles and abstracts by at least two researchers working independently using Abstrackr (Wallace et al., 2012). Based on this screening, 40 reports were selected for full-text screening. Of these studies, 12 were selected for inclusion (see Figure 1 for reasons for exclusion). A backwards search of the citations in these 12 reports was conducted but did not yield additional studies. A forwards search of the 12 reports yielded an additional 8 reports. The citations of previous reviews were examined (Pashler et al., 2008; Cuevas, 2015; Aslaksen and Lorås, 2018; Dinsmore et al., 2022), which yielded one more report. This led to a total of 21 reports of 21 independent studies in this meta-analysis.

### Coding

To prepare the studies for analyses, two researchers coded the methodological and bibliographic information about each study (see Table 1; *κ* = 0.89). Specifically, the sample, study design, learning styles examined, measures of learning, content of instruction/materials, and assessment were recorded to describe studies (see Appendix A for codebook). Study quality was determined based on What Works Clearinghouse (2022) criteria and categorized as meeting standards, meeting standards with reservations, or not meeting standards (see Appendix A for details). Based on these standards, a study must be a randomized experiment to meet standards (although not all randomized experiments meet standards). In randomized experiments, the chance of students being the control or intervention should be equal. In contrast, quasi-experiments involve naturally occurring groups, typically classes in educational research, or controls matched through propensity score matching or regression discontinuity design. Whether a study had randomization (for between subjects) or counterbalancing (for within subjects) is noted in the summary of studies in Table 1. Other study quality criteria such as the face validity for each outcome measure, reliability standards for each outcome measure, and whether there was consistent data collection across conditions are reported in Appendix Table B1. As can be seen in Appendix Table B2, five studies were determined to WWC standards and the remainder did not meet WWC standards.

**Table 1:** Summary of studies.

**Author (date), dissemination type**	**Participants**	**Design**	**Learning styles**	**Neutral/mixed groups**	**Learning activity (instruction/material) and assessment**
Aslaksen and Lorås (2019), journal article	22 college students (average age 22.1 years)	Between subjects (randomly assigned); laboratory study	Visual and auditory (identified and recruited to participate in an earlier prior study based on the Learning Style Survey [LSS], Cohen et al., 2019), completed prior to learning activity	Only students clearly identified as visual or auditory learners on the LSS were eligible to participate	History lesson in audio or text form, assessed using a multiple-choice recall test (written)
Burns (n.d.), undergraduate capstone thesis	37 undergraduates (average age 21.8 years)	Within-subjects (counterbalanced); laboratory study	Auditory and visual (based on cutoff scores from the Styles of Processing Scale, Childers et al., 1985), completed prior to learning activity	None	History lectures with audio only and video, assessed using verbatim fill-in-the-blank items (written)
Chen (2020), journal article	75 university students (between 19 and 25 years old)	Between subjects (randomly assigned); participation was outside of coursework	Read/write and auditory styles (highest score on the visual, auditory, read/write, kinesthetic, VARK questionnaire; Fleming, 2001), completed prior to learning activity	Not mentioned	Web-based instructions on how to use an electronic pen tool with audio narration or onscreen text, assessed on pen skills (kinesthetic)
Chen and Sun (2012), journal article	139 fifth-grade students	Between subjects (randomly assigned); study participation was during the school day	Verbal and visual (based on the Styles of Processing Scale, Childers et al., 1985), completed after learning activity	Not mentioned	Multimedia material on energy education with text (matched to verbal), video, or animation (both matched to visual) conditions, assessed using multiple-choice questions (written)
Chui et al. (2021), journal article	18 trainee pilots (average age 21.89 years)	Within-subjects (counterbalanced); recruited from flight training, but participated outside of course requirements	Visual and auditory styles (based on whether their visual or auditory score on the VARK questionnaire was higher), completed measure prior to learning activity	Participants with equal scores on visual and auditory preferred learning styles were excluded (exact number not stated)	Visual or auditory feedback on flight simulator performance, assessed through follow-up flight performance (kinesthetic)
Cuevas and Dawson (2018), journal article	183 undergraduate and graduate students (between 19 and 50 years old)	Between subjects (randomly assigned); laboratory study	Visual and auditory (highest score on the VARK questionnaire, Fleming and Mills, 1992), completed prior to learning activity	Participants with equivalent visual and auditory scores were excluded (*n* = 21)	20 statements with instructions to either visualize statements (visually matched) or consider pronouncing statements (auditory matched) answering questions about the statements from memory (written)
Ge (2021), journal article	140 college students	Between subjects (randomly assigned); part of course instruction across multiple units	Visual and auditory (Perceptual Learning Style Preference Questionnaire, Reid, 1987), completed prior to learning activity	Participants who were not categorized as a visual or auditory style based on questionnaire scores were excluded from the analyses	Web-based modules on grammar with narration or on-screen text, assessed using multiple-choice questions (written)
Hazra et al. (2013), conference proceedings	139 graduate students	Between subjects (randomly assigned); laboratory study	Visual and verbal (based on the Index of Learning Styles scores, Felder and Soloman, 1997), it is unclear whether this was completed prior to or after the learning activity	Some participants were categorized as neutral and analyzed separately	History and engineering modules with either visual or verbal modes, assessed using gain scores subtracting pretest from posttest scores on recall, recognition, comprehension, and transfer (written)
Kam et al. (2020), journal article	60 English as a foreign language college students (average age 21.5)	Between subjects (randomly assigned); laboratory study	Visual and auditory (based on higher scores on the Caption Reliance Test, Leveridge and Yang, 2014), completed prior to learning activity	Not described	Video lecture on leadership with and without captions, assessed using multiple-choice questions (listening)
Kassaian (2007), journal article	66 university students	Within-subjects (counterbalanced); part of instruction	Visual and auditory (based on scores of both the VAK, Chislet and Chapman, 2005, and a researcher-made self-report), it is unclear whether this was completed prior to or after the learning activity	Participants who did not have consistent results on the two measures were not included (*n* = 3)	New vocabulary words either listened to or viewed, assessed through multiple-choice recognition questions and recall of words (both written)
Lehmann and Seufert (2020), journal article	42 university students (average age 22.55 years)	Between subjects (randomly assigned); laboratory study	Auditory and visual modality preferences (only those who had one modality scoring in the top third and the other modality in the bottom third of scores were included), completed prior to the learning activity	A pool of 223 students was used to select students who had high scores in one modality and low scores in another modality, those not selected were excluded from the study	A scientific text on volcanos either read or listened to, the recall was assessed by literal multiple-choice questions, and comprehension was assessed by open-response questions (written)
Moser and Zumbach (2018), journal article	124 or 113 (depending on analyses) university students (average age 25.17 years)	Between subjects (randomly assigned); laboratory study	Verbal and visual (based on Verbal-Visual Learning Styles Questionnaire and Santa Barbara Learning Styles Questionnaire, Mayer and Massa, 2003 subscales scores, analyzed separately), completed prior to learning activities	Participants who were not categorized as visual or verbal style based on questionnaire scores were excluded from analyses	Multimedia material on plate tectonics with mostly pictures (visual) and mostly text (verbal) conditions, assessed by multiple choice and open-response questions (written, no mention of visuals)
Moussa-Inaty et al. (2019), journal article	61 undergraduate students (between 19 and 30 years old)	Between subjects (quasi-experiment with groups based on styles); laboratory study	Visual and auditory (based on VAK learning style inventory scores), completed prior to learning activity	Students who scored highest as kinesthetic learners were not included	Lessons on lightning with auditory or written conditions, assessed through short answer comprehension questions (written)
Mujtaba et al. (2022), journal article	80 English as a Second Language students (average age 18 years)	Between subjects (quasi-experiment, group assignment decision not stated); part of classroom instruction across multiple sessions	Auditory and read/write (highest score on the VARK questionnaire, Fleming and Mills, 1992), completed prior to learning activity	Some participants were excluded because their auditory or read/write learning style scores were not very high (*n* = 30)	Audio and text-based instruction on grammar, assessed through oral production (aural) and writing tasks (written)
Papanagnou et al. (2016), journal article	162 medical students	Between subjects (randomly assigned); part of medical training	Auditory, visual, and kinesthetic (based on self-report of perceived learning style), completed prior to learning activity	Participants who reported multiple perceived learning style modalities were analyzed separately	Students were trained individually on intravenous (IV) needle placement by instructors using verbal instructions, guiding the hands of the students, or visually demonstrating, assessed through successful IV placement on the first attempt (kinesthetic)
Rassaei (2018), journal article	62 English as a Foreign Language students (between 21 and 37 years old)	Between subjects (randomly assigned); part of classroom instruction in a single session	Auditory and visual (based on scores above the mid-cutoff point on a learning styles questionnaire, Slack and Norwich, 2007), completed prior to learning activity	Participants who scored above the cutoff point for both auditory and visual styles were excluded from the study (exact number not stated)	Reading passages with glosses for new vocabulary (definitions appeared when cursors were hovered over the words) that were in either text or audio, assessed through vocabulary production and recognition (written)
Rassaei (2019), journal article	61 English as a Foreign Language students (between 18 and 37 years old)	Between subjects (randomly assigned); part of classroom instruction in a single session	Auditory and read/write based on the VARK questionnaire (Fleming and Mills, 1992)	Participants who could not be assigned as auditory or read/write styles were excluded from the study (*n* = 65)	Corrective feedback on English article usage that is audio (for auditory style) or text (for read/write style), assessed through an oral test of article usage.
Riding and Douglas (1993), journal article	40 15–16 year old high school students	Between-subjects (randomly assigned); participation was part of the school day	Verbalizers and imagers (based on categorization from the Cognitive Styles Analysis, Riding, 1991), completed after the learning activity	There was an intermediate group analyzed separately (*n* = 19)	Tutorial on car brake systems either only text (matched with verbalizers) or text plus pictures (matched to visualizers), assessed through short recall questions, an explanation question, a problem-solving question, and labeling questions (written).
Rogowsky et al. (2015), journal article	41 adults (between 25 and 40 years old)	Between subjects; laboratory study	Visual and auditory learning styles (based on categorization by the Building Excellence Online Learning Styles Assessment Inventory; Rundle and Dunn, 2010)	Participants with similar preferences for visual and auditory modality were excluded (*n* = 53)	Reading or listening to passages from a history book, assessed using written multiple-choice questions (written).
Rogowsky et al. (2020), journal article	34 fifth-grade students	Within subjects; students participated during the school day	Visual and auditory styles (categorized by the Learning Styles: The Clue to You! measure), completed after learning activity	Participants with similar preferences for visual and auditory modality were excluded (*n* = 73)	Reading or listening to texts from a standardized comprehension test, assessed based on written test items.
Tadayonifar et al. (2021), journal article	13 English as a Foreign Language students (ages 19–20)	Within-subjects (orders randomly assigned); students participated as part of class activities	Read/write and auditory styles (based on the highest scores on the VARK learning style inventory, Fleming, 2001), completed prior to learning activity	Participants with similar scores were identified as mixed styles	Reading passages with glosses for new vocabulary (definitions appeared when cursors were hovered over the words) that were either in text or audio, assessed through vocabulary test

The number of participants in this table is the number of participants in the analytic sample used to calculate effect sizes in the meta-analysis. Several studies excluded participants because of their learning style scores or had conditions unrelated to the research questions of this meta-analysis and subsequently had larger samples than are reported in this table. Some studies did not cite the authors of the learning styles measure they used and only provided the name of the measure.

### Statistical procedures

**Figure 2:**
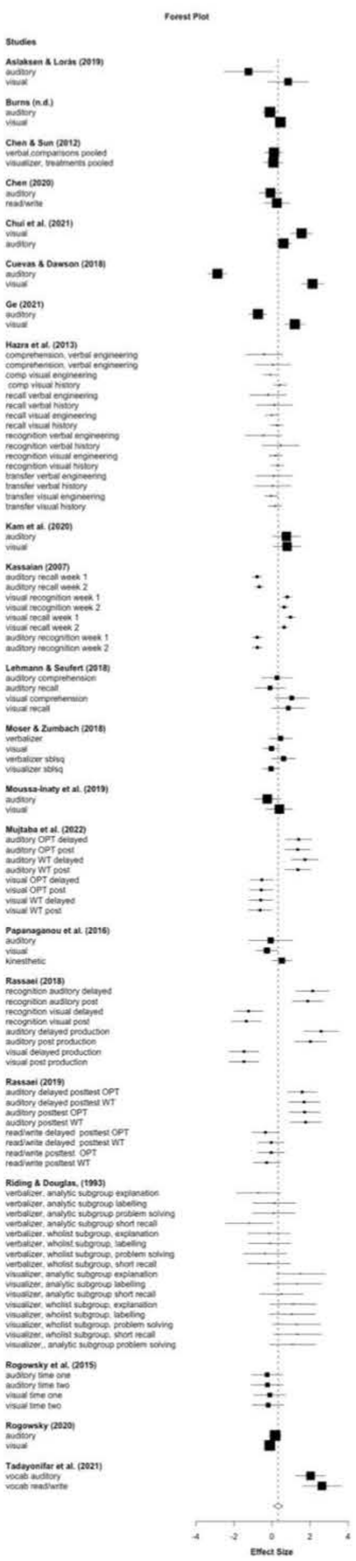
Forest plot of effect sizes.

The effect sizes for each learning outcome comparing matched and unmatched instruction were calculated. Hedges’ *g* was used as an effect size calculated using Meta-Essential tools (Suurmond et al., 2017). A positive Hedges’ *g* indicates better learning outcomes for matched than unmatched instruction. To account for multiple effect sizes within each study, a robust variance estimation (RVE) was used. An RVE is a statistical technique that accounts for dependencies within studies while still allowing for the unique contribution of each effect size to be considered (Tanner-Smith et al., 2016). Each of the study effect sizes is shown in Table 2, and a forest plot is in Figure 2. Learning outcomes indicating a crossover interaction as articulated in Pashler et al. (2008) in which at least two styles had higher learning outcomes with matched instruction are bolded in Table 2.

**Table 2:** Effect sizes with variances and number of participants.

**Study, learning style group, condition (if more than one), measure (if more than one), subgroups (if any)**	**Number of participants**	**Hedges’ *g***	**Variance of Hedges’ *g***
Aslaksen and Lorås (2019), auditory	9	−1.25	0.44
Aslaksen and Lorås (2019), visual	13	0.85	0.30
Burns (n.d.), auditory	7	−0.10	0.04
Burns (n.d.), visual	30	0.44	0.01
Chen (2020), auditory	41	−0.08	0.09
Chen (2020), read/write	34	0.26	0.11
**Chui et al. (** **2021)** **, auditory**	9	1.56	0.09
**Chui et al. (** **2021)** **, visual**	9	0.61	0.04
**Chen and Sun (** **2012)** **, verbal, interaction comparison**	73	0.11	0.06
**Chen and Sun (** **2012)** **, visualizer, interactive treatment**	66	0.08	0.07
Cuevas and Dawson (2018), auditory	118	−2.87	0.07
Cuevas and Dawson (2018), visual	65	2.13	0.10
Ge (2021), auditory	76	−0.74	0.06
Ge (2021), visual	64	1.20	0.07
Hazra et al. (2013), verbal, engineering comprehension	15	−0.41	0.24
**Hazra et al. (** **2013)** **, verbal, history comprehension**		0.06	0.24
Hazra et al. (2013), verbal, engineering recall		−0.21	0.24
**Hazra et al. (** **2013)** **, verbal, history recall**		0.13	0.24
Hazra et al. (2013), verbal, engineering recognition		−0.44	0.24
**Hazra et al. (** **2013)** **, verbal, history recognition**		0.46	0.24
Hazra et al. (2013), verbal, engineering transfer		0.10	0.24
**Hazra et al. (** **2013)** **, verbal, history transfer**		0.04	0.24
Hazra et al. (2013), visual, engineering comprehension	124	−0.10	0.03
**Hazra et al. (** **2013)** **, visual, history comprehension**		0.43	0.03
Hazra et al. (2013), visual, engineering recall		0.00	0.03
**Hazra et al. (** **2013)** **, visual, history recall**		0.25	0.03
Hazra et al. (2013), visual, engineering recognition		0.19	0.03
**Hazra et al. (** **2013)** **, visual, history recognition**		0.30	0.03
Hazra et al. (2013), visual, engineering transfer		−0.04	0.03
**Hazra et al. (** **2013)** **, visual, history transfer**		0.16	0.03
**Kam et al. (** **2020)** **, auditory**	29	0.76	0.14
**Kam et al. (** **2020)** **, visual**	31	0.79	0.13
**Kassaian (** **2007)** **, auditory, week 1**	29	0.78	0.02
**Kassaian (** **2007)** **, auditory, week 2**		0.77	0.02
**Kassaian (** **2007)** **, visual, week 1**	37	0.80	0.01
**Kassaian (** **2007)** **, visual, week 2**		0.64	0.02
**Lehmann and Seufert (** **2020)** **, auditory comprehension**	21	0.26	0.18
Lehmann and Seufert (2020), auditory recall		−0.11	0.18
**Lehmann and Seufert (** **2020)** **, visual comprehension**	21	1.04	0.20
Lehmann and Seufert (2020), visual recall		0.86	0.19
Moser and Zumbach (2018), verbalizer VVQ	42	0.45	0.09
Moser and Zumbach (2018), verbalizer SBLSQ	40	0.61	0.10
Moser and Zumbach (2018), visualizer VVQ	82	−0.03	0.05
Moser and Zumbach (2018), visualizer SBLSQ	73	−0.04	0.05
Moussa-Inaty et al. (2019), auditory	31	−0.25	0.12
Moussa-Inaty et al. (2019), visual	30	0.39	0.13
Mujtaba et al. (2022), auditory OPT delayed	40	1.40	0.12
Mujtaba et al. (2022), auditory OPT post		1.35	0.12
Mujtaba et al. (2022), auditory WT delayed		1.73	0.13
Mujtaba et al. (2022), auditory WT post		1.36	0.12
Mujtaba et al. (2022), visual OPT delayed	40	−0.53	0.10
Mujtaba et al. (2022), visual OPT post		−0.56	0.10
Mujtaba et al. (2022), visual WT delayed		−0.59	0.10
Mujtaba et al. (2022), visual WT post		−0.63	0.10
Papanagnou et al. (2016), auditory	52	−0.07	0.34
Papanagnou et al. (2016), kinesthetic	62	0.52	0.07
Papanagnou et al. (2016), visual	48	−0.27	0.08
Rassaei (2018), auditory delayed production	32	2.58	0.22
Rassaei (2018), auditory delayed recognition		2.14	0.19
Rassaei (2018), auditory post production		2.02	0.18
Rassaei (2018), auditory post recognition		1.88	0.17
Rassaei (2018), visual delayed production	30	−1.47	0.16
Rassaei (2018), visual delayed recognition		−1.23	0.15
Rassaei (2018), visual post production		−1.48	0.16
Rassaei (2018), visual post recognition		−1.35	0.16
Rassaei (2019), auditory delayed OPT	31	1.59	0.16
Rassaei (2019), auditory post OPT		1.72	0.17
Rassaei (2019), auditory delayed WT		1.69	0.17
Rassaei (2019), auditory post WT		1.77	0.17
Rassaei (2019), read/write delayed OPT	30	−0.34	0.13
Rassaei (2019), read/write post OPT		−0.04	0.13
Rassaei (2019), read/write delayed WT		−0.04	0.13
Rassaei (2019), read/write post WT		−0.28	0.13
Riding and Douglas (1993), verbalizer, analytic subgroup explanation	10	−0.70	0.35
Riding and Douglas (1993), verbalizer, analytic subgroup labelling		0.12	0.33
Riding and Douglas (1993), verbalizer, analytic subgroup problem solving		0.08	0.33
Riding and Douglas (1993), verbalizer, analytic subgroup short recall		−1.20	0.40
Riding and Douglas (1993), verbalizer, wholist subgroup, explanation	10	−0.15	0.33
Riding and Douglas (1993), verbalizer, wholist subgroup, labeling		−0.11	0.33
Riding and Douglas (1993), verbalizer, wholist subgroup, problem solving		−0.37	0.33
Riding and Douglas (1993), verbalizer, wholist subgroup, short recall		−0.17	0.33
Riding and Douglas (1993), visualizer, analytic subgroup explanation	10	1.51	0.44
Riding and Douglas (1993), visualizer, analytic subgroup labeling		1.33	0.41
Riding and Douglas (1993), visualizer, analytic subgroup problem solving		1.08	0.38
Riding and Douglas (1993), visualizer, analytic subgroup short recall		0.50	0.34
Riding and Douglas (1993), visualizer, wholist subgroup, explanation	10	1.11	0.39
Riding and Douglas (1993), visualizer, wholist subgroup, labeling		1.05	0.38
Riding and Douglas (1993), visualizer, wholist subgroup, problem solving		1.30	0.41
Riding and Douglas (1993), visualizer, wholist subgroup, short recall		1.35	0.42
Rogowsky et al. (2015), auditory time one	21	−0.25	0.18
Rogowsky et al. (2015), auditory time two		−0.24	0.18
Rogowsky et al. (2015), visual time one	20	−0.11	0.18
Rogowsky et al. (2015), visual time two		−0.20	0.18
Rogowsky et al. (2020) auditory	12	0.17	0.03
Rogowsky et al. (2020) visual	22	−0.12	0.02
**Tadayonifar et al. (** **2021)** **, auditory**	7	2.03	0.16
**Tadayonifar et al. (** **2021)** **, read/write**	6	2.63	0.27

Learning outcomes indicating a crossover effect as articulated in Pashler et al. (2008) in which at least two styles had higher learning outcomes with matched instruction are bolded.

## Results

The overall main effect of matching instruction to learning styles on learning outcomes was estimated using RVE based on 21 studies and 101 effect sizes and assumed dependency (intercorrelation of dependent effects within studies) of ρ = 0.8. The findings indicated an overall positive effect on learning outcomes for matching instruction to learning styles compared to unmatched instruction, *g* = 0.32, SE = 0.12, 95% CI = [0.07, 0.57], *p* = 0.01. There was substantial variability with a τ^2^ of 0.77 and *I^2^* of 91.17. A sensitivity analysis was conducted with a range of dependent effect size correlations. As can be seen in Table 3, the effect was consistent across assumed dependencies.

**Table 3:** Sensitivity analyses for the assumed dependency of effect sizes.

	Rho = 0	Rho = 0.2	Rho = 0.4	Rho = 0.6	Rho = 0.8	Rho = 1
Hedges’ *g*	0.33	0.32	0.32	0.32	0.32	0.32
Std. error	0.12	0.12	0.12	0.12	0.12	0.12
Tau.sq	0.77	0.77	0.77	0.77	0.77	0.78

### Publication bias

**Figure 3:**
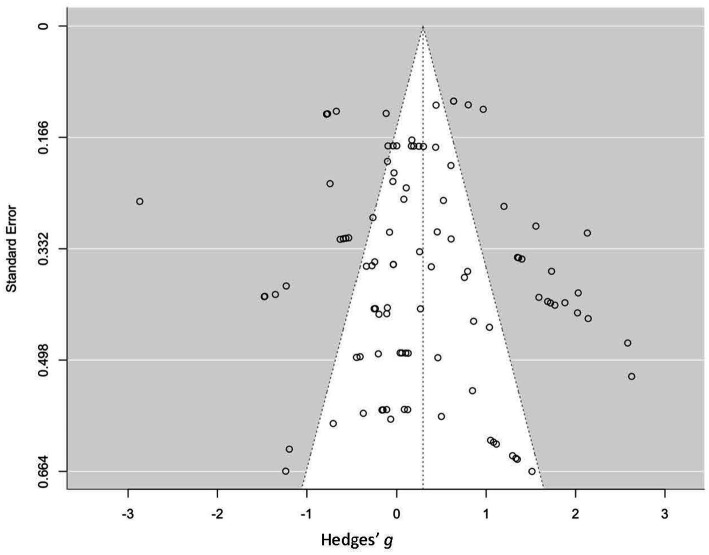
Funnel plot of effect sizes.

Publication bias was examined to see whether there was overreporting of positive effects. A funnel plot was generated using the “metafor” package in R (Viechtbauer, 2010; see Figure 3). Based on a visual inspection of the funnel plot, the distribution of effect sizes was approximately symmetrical with smaller and larger studies having similar distances away from the mean (indicated by the vertical line; Lin and Chu, 2018). Egger’s test of the intercept was not significant, *b* = −0.058, 95% CI [−0.52, 0.41], *p* = 0.11. Taken together, the funnel plot and Egger’s test indicate that publication bias does not appear to be the reason for the positive effect of matching instruction to learning styles.

### Crossover interactions

The number of crossover interactions (at least two styles benefited from matched instruction within a learning outcome measure) was calculated. Based on the tally of the bolded effect sizes in Table 2, there are 11 learning outcome measures in which matched instruction benefited at least 2 learning styles as indicated by Hedges’ *g* greater than 0. This was out of a total of 42 learning outcome measures that were compared indicating that 26.19% had the type of crossover interaction necessary to support the meshing hypothesis as articulated by Pashler et al. (2008).

As indicated in Figure 1, five that had their full texts screened did not have sufficient statistics to calculate the effect sizes reported. In addition, seven reports identified through other searches did not have sufficient statistics to calculate effect sizes but otherwise met inclusion criteria. Based on the descriptions of the findings of these 12 studies in Tables 3, 4 of these studies indicated a crossover interaction (25.00%). Therefore, the findings from the studies without sufficient statistics reported appear to be similar to the studies included in the meta-analysis in terms of crossover interactions.

**Table 4:** Studies without sufficient descriptive statistics reported to calculate effect sizes.

**Author (date), dissemination type**	**Description of findings**
Bareither et al. (2013), journal article	The read/write group scored higher when learning through modules (matched) than making clay models (unmatched). There were no differences for the matched (clay models) and unmatched (modules) kinesthetic groups.
**Fajari et al. (** **2020)** **, journal article**	**There was a significant interaction indicating a crossover effect in which both style groups benefited from instruction matched to their styles.**
**Höffler and Schwartz (** **2011)** **, journal article**	**There was a significant interaction between styles and conditions, which resulted in a crossover as indicated in the figure indicating a benefit of matching instruction to style.**
**Huang (** **2019)** **, journal article**	**The matched conditions had higher scores on learning outcomes than the unmatched conditions, but this was not significant and the interaction between styles and condition was not reported.**
Koć-Januchta et al. (2019), journal article	No significant interaction between style and condition. Correlations with style scores, not learning style groups, were examined.
Koć-Januchta et al. (2020), journal article	There appeared to be a benefit of matching instruction for visualizer, but not for verbalizers. Correlations with style scores, not learning style groups, were examined.
Kollöffel (2012), journal article	Interactions between styles and conditions were not reported.
Kraemer et al. (2017), journal article	The interaction between conditions and style was not significant. The direction of the effect of matched compared to unmatched instruction within style group could not be determined from the article.
Massa and Mayer (2006), Exp 1 and Exp 2, journal article	No significant interactions between style and condition for either experiment. The direction of the effect of matched compared to unmatched instruction within style group could not be determined from the article.
**Riding and Ashmore (** **1980)** **, journal article**	**The interaction between styles and condition was significant. The matched conditions had higher scores on learning outcomes than the unmatched conditions (crossover).**
Sankey et al. (2011)	Performance on learning outcomes by style groups and conditions was not reported.
Thomas and McKay (2010)	Significant interactions between styles and conditions were reported. Styles were not grouped, but the regression coefficients indicated a crossover effect in which style scores positively predicted learning outcomes in matched conditions, but not unmatched conditions.

Bold indicates that the findings of the study indicated a crossover effect.

### Moderator analysis

To estimate whether these potential moderators varied the effect of matching instruction to learning style, the package “robumeta” in R was used (Fisher and Tipton, 2015). The study design (between or within subjects), modality of instruction/materials (visual, verbal, or auditory), whether the assessment was in the same modality as the instruction, and study quality (does not meet WWC standards or meets WWC standards) were all coefficients estimated in the meta-regression model. For consistency across studies, “visual” matched instruction that was text-based was coded as “verbal or read/write.” Based on the output of the meta-regression model, none of the moderators were significant (see Table 5). Therefore, it is unclear what the source of variability in the aggregate findings is.

**Table 5:** Meta-regression model.

	Beta	SE	*t*-value	Dfs	*p*	95% CI Lower	95 CI Upper
Intercept	0.55	0.35	1.57	15.81	0.14	−0.20	1.30
Within or between	0.49	0.41	1.18	6.68	0.28	−0.50	1.47
Style	−0.25	0.26	−0.97	12.31	0.35	−0.82	0.31
Assessment	−0.18	0.24	−0.76	18.57	0.46	−0.69	0.32
Study quality	0.22	0.36	0.61	8.88	0.56	−0.60	1.04

Within-subjects studies were coded 0, and between-subjects studies were 1. Style was coded 0 for visual, 1 for verbal, and 2 for auditory. Assessment was coded 0 if the instruction and assessment were in the same modality and 1 if they were in different modalities. Study quality was coded 0 for not meeting What Works Clearinghouse Standards and 1 for meeting standards. SE, standard error; DFs, degrees of freedom.

### Sensitivity analysis

A sensitivity analysis was conducted to examine whether altering the inclusion criteria changed the results. There was only one study in which instruction was adopted to a kinesthetic learning style (Papanagnou et al., 2016). Removing this study from the RVE analyses indicated an overall aggregated benefit of matched instructions to learning styles compared to unmatched, *g* = 0.34, SE = 0.13, 95% CI = [0.08, 0.60], *p* = 0.01 with 20 studies and 98 effect sizes. This finding is similar to the findings when the kinesthetic learning intervention was included. There were two studies that were quasi-experiments without random assignment (Moussa-Inaty et al., 2019; Mujtaba et al., 2022). This was a concern given that they did not demonstrate baseline equivalence in the study quality coding (see Appendix Table B1). Therefore, an RVE was conducted with the two quasi-experiments removed from the analyses. The results of the RVE were similar to the quasi-experiments removed in that there was an overall aggregated benefit of matched instruction to learning styles compared to unmatched, *g* = 0.33, SE = 0.13, 95% CI = [0.05, 0.61], *p* = 0.02 with 19 studies and 91 effect sizes.

## Discussion

Educational researchers consider the concept of better learning through matching instruction to learning styles to be a neuromyth that completely lacks empirical evidence (Brown, 2023). However, the findings from this meta-analysis indicated a small, but statistically reliable benefit of matching instruction based on learning styles. This aligns with the majority of educators’ perspectives (Dekker et al., 2012; Nancekivell et al., 2020; Eitel et al., 2021) but conflicts with the conclusions of previous reviews by educational researchers (Cuevas, 2015; An and Carr, 2017; Aslaksen and Lorås, 2018; Dinsmore et al., 2022; Yan and Fralick, 2022). What distinguishes this meta-analysis from previous reviews is (1) its singular focus on studies comparing instruction matched and unmatched to modality learning styles and (2) its systematic approach to gathering relevant studies and aggregating findings. The lack of evidence noted in previous reviews may be due to a lack of power in individual studies. The cumulative evidence of aggregated effects appeared to have sufficient power to detect an effect. However, the majority of learning outcomes did not indicate a crossover interaction that would validate accommodation to learning styles. However, a non-trivial minority of learning outcomes did indicate the crossover interaction indicative of supporting the matching hypothesis based on Pashler et al. (2008). An important caveat is that most of the studies indicating a crossover interaction did not meet quality standards as determined by the What Works Clearinghouse (2022). Taken together, these findings may be interpreted that it is too much of an overreach to insist learning styles should be incorporated into instructional practices.

Given the time and resources required for matching instruction to learning styles coupled with the potential for harm through psychological essentialism (Vasquez, 2009; Fallace, 2019, 2023a,b; Nancekivell et al., 2020; Sun et al., 2023), we stated in the literature review that accommodating instruction to learning styles would need to have substantial benefits to mitigate their potential for harm. To consider this issue, it may be helpful to compare the effect size noted in this meta-analysis (Hedges’ *g* = 0.32) with those from other methods of adapting instruction. For example, the *modality effect*, in which listening to verbal information while viewing visual representations, rather than reading the same verbal information alongside visuals has Hedges’ *g* of 0.70 (Noetel et al., 2022); that is, the benefit of listening, rather than reading verbal information that accompanies visual representations across all students, appears to have twice the effect than was noted in this meta-analysis matching instruction to learning styles and would be less time-consuming and expensive to implement. Removing interesting or irrelevant information included with the lesson has Hedges’ *g* of 0.33 (Sundararajan and Adesope, 2020). Segmenting instruction into meaningful learner-paced units has a benefit of Hedges’ *g* of 0.32 compared to continuous information (Rey et al., 2019). Finally, an overall application of multimedia principles to learning has Hedge’s *g* of 0.28 (Noetel et al., 2022).

When examining an overview of meta-analysis on multimedia design for learning, accommodating instruction based on learning styles in the current meta-analysis is generally about the same size or smaller than various multimedia designs (e.g., signaling important information, animation, and pleasant colors/anthropomorphic; Noetel et al., 2022). However, all of the multimedia design principles reviewed involved having students each receive the same instructional changes, whereas accommodating instruction based on learning styles by definition involves at least two types of instruction (Noetel et al., 2022). In addition, 85% of the studies in the current meta-analysis did not include all participants in the final sample because their learning styles scores did not allow for confident categorization and matching/unmatching to instructional modality. Therefore, we, the authors, deeply question whether the found benefits of learning styles in this meta-analysis warrant accommodating instruction, especially for widespread use. Based on previous studies, well-structured instructional design may be more effective across all students and would involve less time than accommodating to learning styles.

Participant expectations may be relevant to interpreting the findings from the studies in this meta-analysis (Vasquez, 2009; Sun et al., 2023). Generally, participants were asked about their modality preferences and then engaged in a learning activity shortly thereafter (only three studies specifically stated participants were asked to complete learning style measures after the learning activity; see Table 1). If participants were aware of their learning styles prior to engaging in a task that matched or unmatched their style, they may have had different expectations for success and engagement that affected their learning (Vasquez, 2009). For example, one study categorized students based on fake/induced learning styles (Moser and Zumbach, 2015). In other words, students took a learning styles assessment and were told (incorrectly) that they scored as visualizers or verbalizers. Students scored higher when their instructional materials “matched” their fake/induced learning style compared to the unmatched conditions, but there were no benefits to matching based on their actual categorizations based on the learning styles assessment (Moser and Zumbach, 2015). This is described in the *learning styles genesis model* in which appraisal and decision processes based on external feedback about learning styles along with previous experiences with modalities shape learning outcomes (Moser and Zumbach, 2018). Moreover, participants may have had a situational interest in the content triggered by immediately receiving instruction in their stated preferences (Bernacki and Walkington, 2018). This likely would not continue long term as maintained interest requires a personal connection (Høgheim and Reber, 2017).

Learning styles may be conflated with modality-specific skills (An and Carr, 2017). In other words, participants may simply be better at reading if they indicate a read/write style or listening if they indicate an auditory style. This results in a *jangle fallacy* in which two similar constructs (e.g., modality skill and learning styles) are considered different because they have different terminology (Kelley, 1927; Beisley, 2023). It should be noted, however, that if this is the case, skills in a modality may have been developed because of preferences in that modality, which would, in turn, lead to more practice and more skill in a particular modality. It would be extremely difficult to disentangle the initiating factor in this (hypothetical) perpetual cycle of skill and preference. However, more inquiry into fake/induced learning styles such as that by Moser and Zumbach (2015) would be a useful means of testing whether the skill is confounded with style given that fake/induced styles would be randomly assigned and subsequently modality skills should be similar across “styles.”

A key feature of the matching hypothesis is a crossover interaction in which matched instruction benefits learning for only the group for which it is matched. This matched instruction differs depending on the learning style of the student. In the current meta-analysis, approximately one-fourth of the learning measures indicated a crossover interaction in which there were positive effect sizes for matched instruction for two different learning styles (Kassaian, 2007; Chen and Sun, 2012; Hazra et al., 2013; Kam et al., 2020; Lehmann and Seufert, 2020; Chui et al., 2021; Tadayonifar et al., 2021). This raises the question of what characteristics of these studies and learning measures may be responsible for the crossover interaction. However, these studies are quite heterogeneous. Samples include young adult college students, elementary school students, and aircraft pilot trainees. The learning styles and their inventories varied and included the Styles of Processing Scale (Childers et al., 1985), VARK questionnaire (Fleming, 2001), Index of Learning Styles Scores (Felder and Soloman, 1997), and Caption Reliance Test (Leveridge and Yang, 2014). Content and learning measures were also varied such as memory and recall of history (Hazra et al., 2013), multiple-choice questions about energy education (Chen and Sun, 2012), and flight simulator performance (Chui et al., 2021). Therefore, there does not seem to be any consistent feature across these studies based on the information coded for this meta-analysis that would elucidate the mechanism behind the crossover interaction. Subsequently, the lack of understanding of what circumstances could foster a crossover interaction is an additional reason for caution in implementing the matched instruction for learning styles. Without knowing what features are conducive to effective matched instruction, it is extremely difficult to have effectively matched instruction across identified learning styles.

### Implications

We advise extreme caution if using the findings from this meta-analysis to justify matching instruction to learning styles. If choosing to incorporate learning styles, then learning styles should never be ascribed as a feature of a cultural group, especially by individuals outside of that group, as this leads to unwarranted and potentially harmful expectations based on group membership (Gutiérrez and Rogoff, 2003; Fallace, 2023a,b). Moreover, learning style interventions are costly in terms of both time and money (Pashler et al., 2008). By definition, matching instruction based on learning styles requires multiple versions of instruction or materials to be developed.

If learning styles are incorporated into education, we strongly recommend that they be implemented in the context of multimodality for learning. By providing information in more than one modality, such as text with visuals, the same materials could arguably appeal to both verbal and visual learning styles while grounded in theories of human cognition such as dual coding (Noetel et al., 2022). Engaging multiple senses is generally beneficial for learning (Nguyen et al., 2022). In addition, providing students with audio-assisted text may also be beneficial, particularly for learning beyond one’s native language (Clinton-Lisell, 2023a), and logically appeal to auditory and verbal preferences. Not only is multimodality known to be effective for learning but even individuals with strong essentialist beliefs about learning styles support multimodal learning as effective (Nancekivell et al., 2021). Moreover, offering multiple modalities for learning provides an inclusive education for students with perceptual disabilities to have access to the content (Thomas et al., 2015; Griful-Freixenet et al., 2017).

### Limitations and future directions

Limitations to the studies were included in the meta-analysis. As indicated in the study quality coding, the majority of the outcome measures did not have reliability metrics reported. The lack of information about reliability, as noted in the study quality scoring, leads to challenges in determining the validity of the findings. Indeed, the primary issue with study quality is due to an inability to assess reliability due to a lack of reporting across multiple studies. Unfortunately, a lack of reporting reliability statistics is a common issue across multiple social science and education disciplines (Barry et al., 2014; Lovejoy et al., 2014; Han, 2016; Parsons et al., 2019; Flake, 2021). This illustrates the need to ensure that reliability is reported throughout the peer review and publication process. Indeed, publication reporting standards in psychology, through the American Psychological Association (Appelbaum et al., 2018), state that the reliability of measures should be reported.

The studies were all single sessions in duration and subsequently claims about long-term effects cannot be determined from the meta-analysis. Moreover, there was substantial variation in the findings across outcomes that was not explained in the meta-regression. This could be due to insufficient power to identify moderators in the meta-regression analyses (Schmidt, 2017). Furthermore, the studies were limited to those disseminated in English due to the linguistic limitations of the research team. It is possible the inclusion of more languages would have led to different outcomes. In addition, all but two of the reviewed studies were from journal articles. Although the publication analyses did not indicate publication bias, it is still an issue to consider given that only two studies were from the gray literature in which non-significant findings are more likely to be reported (Cairo et al., 2020). There is also possible bias when considering studies as several authors were contacted with requests for data to calculate effect sizes, but only some of the authors provided this information. There may be response bias regarding the findings that were calculated based on author-provided data. However, it should be noted that authors frequently do not respond to requests for data (Tedersoo et al., 2021).

The studies in this meta-analysis all categorized their participants based on learning styles, but the methods of categorization varied. There were a range of measures used and the cutoff for categorization of learning styles differed by study as well. This makes the generalizability of the findings challenging. Moreover, there was substantial variability in the outcome measures. Only 21 studies were identified that met the criteria for testing the matching hypothesis and reported sufficient statistics to conduct effect sizes. In particular, there were not enough studies to examine whether having the learning styles assessment before or after the learning activity varied the findings. This is unfortunate given the concerns about self-fulfilling prophecies and findings from Moser and Zumbach (2015) with fake, induced learning styles.

## Conclusion

Learning styles are a controversial topic in education. In this meta-analysis, we sought to inform the controversy with aggregated findings based on a comprehensive search for studies. An overall small, positive effect was noted. However, this aggregated effect should be interpreted with caution given that most studies did not indicate a crossover interaction. Such a crossover interaction would have been necessary to support the claim that matching instruction to learning styles benefits students from different learning styles. Given the high amount of variability in the findings and infrequent crossover interactions, it is far from conclusive that there is actually a benefit to matching the modality of instruction to students’ learning styles. Teaching with multiple modalities may be preferable to the costly and labor-intensive practice of matching instruction to learning styles given the empirical evidence for benefits across students for multimodal instruction.

## Data availability statement

The datasets presented in this study can be found in online repositories. The names of the repository/repositories and accession number(s) can be found at: https://osf.io/5heum/.

## Author contributions

VC-L: Conceptualization, Data curation, Funding acquisition, Supervision, Writing – original draft, Writing – review & editing. CL: Conceptualization, Data curation, Writing – original draft, Writing – review & editing.
