# Supplementary material for: Is it really a neuromyth? A meta-analysis of the learning styles matching hypothesis
Source: Front Psychol. 2024 Jul 10;15:1428732. doi: 10.3389/fpsyg.2024.1428732 (PMC11270031; doi:10.3389/fpsyg.2024.1428732)
Supplement: Supplementary file 1 [file Data_Sheet_1.docx]

**Appendix A**

**Codebook**

The following descriptive information will be coded about each eligible study. Directions for coding are after each item:

1. Bibliographic information (authors, year). This is included in the report, usually at ethe beginning.
2. Sample size (number of participants). This information is in the Methods, usually when the participants are described.
3. Measures of learning (e.g., multiple choice, open ended, recall). This is usually in the Methods in a Materials or Measures subsection.
4. Statistics for meta-analysis (e.g., means and standard deviations/standard errors; *t*-tests, or *F*-statistics). These should be in the results. If not available in the results, the author needs to be emailed to request the statistics.
5. Grades and age of participants. This is usually in the participants section of the Methods.
6. Study design. Indicate whether the study was between or within subjects and also if there was randomization for between subjects or counterbalancing for within subjects.
7. Classroom or lab. Indicate whether the setting of the study was part of a class/course, part of the school day, or part of a controlled research lab environment.
8. Learning styles. What styles were examined (modality styles only for this review) and how were they assessed (what measure).
9. Timing. Was the learning styles assessment before or after the learning activity (instruction or material).
10. Neutral/mixed groups. Were all participants eligible and grouped into learning styles designations (e.g., verbal, visual, auditory)? If not, indicate if students with mixed or neutral styles were not included in analyses or if they were analyzed separated. In addition, indicate how many participants were considered neutral or mixed styles.
11. Content of the learning activity or lesson. Indicate the topic of the learning activity or lesson and how it was matched to styles.
12. Assessment. Indicate how learning from the activity or lesson content was assessed. For examples, multiple-choice questions, free recall, essay questions.
13. Dissemination. Indicate the venue of publication (journal article, conference presentation/proceeding, dissertation, thesis, book chapter, unpublished data, or other).

For study quality, use the following guidelines. Language taken directly from the handbook:

**Study Quality Coding (from What Works Clearinghouse)**

| **Item** | **Criteria for yes answer** |
| --- | --- |
| 1. Is intervention and comparison group membership determined through a random process (i.e., is it a RCT)? | Group assignment is by chance. |
| 1. Was there evidence of randomization being compromised? | There was evidence that any participants in sample were not randomly assigned to group. There was evidence that participants who intended to receive treatment, but did not receive treatment, were analyzed as comparison group participants (rather than as intent-to-treat). |
| 1. Is the combination of overall and differential attrition high? | Follow Figure II.2 based on percentages of both overall and differential attrition on page 11 of the WWC handbook. Exclusions due to random sampling of subsets of participants or group status inclusion do not count as attrition provided there was no differential removal by group. |
| 1. For studies that are RCTs with high attrition or compromised (answers yes to 2 and 3) or are quasi-experimenters, is equivalence established at baseline for the groups in the sample? | The absolute effect size between groups as baseline was less than .05 or it was between .05 and .25 and statistical adjustment was used. |
| 1. For each outcome, does it have face validity? In other words, does it appear to measure what it is reported to measure? | The outcome appears to measure what it is reported to measure. |
| 1. Does the outcome measure meet minimum reliability statistics? | Internal consistency (e.g., Cronbach’s alpha) of at least .60, temporary stability and test-retest reliability of at least .40, inter-rater reliability (correlation) of at least .50, inter-rater agreement (percent agreement and kappa) of at least .80 (for percent agreement) and at least .60 (for kappa) based on at least 20 percent overlap in judgments |
| 1. Is the outcome measure overly aligned with the intervention? In other words, is there an absence of one condition having an unfair advantage over the other condition? | There were materials or content used in one condition that were not available to the other condition. |
| 1. Was there consistent data collection procedures? Were the data collected in the same manner for all conditions? | Data were collected in the same manner across collection (WWC assumes data were collected in the same manner if no information to the contrary was stated in the report). |
| 1. Were there confounding factors that were always present for one condition and never present for another condition that were unrelated to the condition characteristics? For example, was there an N = 1 confounding factor such as one condition only experiencing one teacher (who wasn’t with the other condition(s). | There was at least one confounding factor. |

**Eligible to meet WWC Group Design Standards without Reservations:** Answer to item 1 is yes and answers to items 2 and 3 are no (item 4 does not apply).

**Eligible to meet WWC Group Design Standards with Reservations:** Answer to item 4 is yes.

**Does Not Meet WWC Group Design Standards:** Answer to item 4 is no.

For items 5-8, “if there is not a single finding that was measured using an outcome measure consistent with the WWC standards or if the study contains a confounding factor that affects all findings, the study will receive a rating of Does Not Meet WWC standards.” In other words, 5, 6, and 7 need to be yes, and items 8 and 9 need to be no.

For item 9, a no would make the study designated “does not meet WWC group design standards.”

What Works Clearinghouse (2022). *WWC Version 5.0 procedures and standards handbook.* <https://ies.ed.gov/ncee/wwc/handbooks#procedures>
