# Supplementary material for: Is it really a neuromyth? A meta-analysis of the learning styles matching hypothesis
Source: Front Psychol. 2024 Jul 10;15:1428732. doi: 10.3389/fpsyg.2024.1428732 (PMC11270031; doi:10.3389/fpsyg.2024.1428732)
Supplement: Supplementary file 2 [file Data_Sheet_2.docx]

**Appendix B**

Study Quality Coding Results

See Appendix A for guidelines

**Table B1**

Study quality coding

| **Study** | **Learning**  **outcome** | **Item 1 (study)** | **Item 2 (study)** | **Item 3 (study)** | **Item 4 (study)** | **item 5 (outcome)** | **item 6 (outcome)** | **item 7 (outcome)** | **item 8 (outcome)** | **item 9 (study)** |
| --- | --- | --- | --- | --- | --- | --- | --- | --- | --- | --- |
| Aslaksen & Loras (2019) | multiple-choice recall | randomized | no | no | n/a | yes | not reported | no | yes | no |
| Burns (nd) | verbatim fill-in-the blank items | counterbalanced | no | no | n/a | yes | not reported | no | yes | no |
| Chen (2020) | transfer | randomized | no | no | n/a | yes | not reported | no | yes | no |
| Chen & Sun (2012) | learning performance | randomized | no | unclear as the analytic sample size differs by measure (likely due to chance attendance that day) | no | yes | not reported | no | yes | no |
| Chui et al. (2021) | flight performance | counterbalanced | no | no | n/a | yes | not reported | no | yes | no |
| Cuevas & Dawson (2018) | recall | randomized | no | no | n/a | yes | Interrater reliability of .99 | no | yes | no |
| Ge (2021) | posttest | randomized | no | no | n/a | yes | not reported | no | yes | no |
| Hazra et al. (2013) | posttest | randomized | no | no | n/a | yes | not reported | no | yes | no |
| Kam et al. (2020) | listening comprehension | randomized | no | no | n/a | yes | not reported | no | yes | no |
| Kassaian et al. (2007) | short term recognition | counterbalanced | no | no | n/a | yes | not reported | no | yes | no |
| Kassaian et al. (2007) | long term recognition | counterbalanced | no | no | n/a | yes | not reported | no | yes | no |
| Kassaian et al. (2007) | short term recall | counterbalanced | no | no | n/a | yes | not reported | no | yes | no |
| Kassaian et al. (2007) | long term recall | counterbalanced | no | no | n/a | yes | not reported | no | yes | no |
| Lehmann & Seufert (2020) | multiple choice questions | randomized | no | no | n/a | yes | not reported | no | yes | no |
| Lehmann & Seufert (2020) | comprehension | randomized | no | no | n/a | yes | not reported | no | yes | no |
| Moser & Zumbach (2018) | knowledge test | randomized | no | no | n/a | yes | not reported | no | yes | no |
| Moussa-Inaty et al. (2019) | short answer | quasi | n/a | no | no | yes | not reported | no | yes | no |
| Mujtaba et al. (2022) | writing | quasi | n/a | no | no | yes | yes (ICC = .82) | no | yes | no |
| Mujtaba et al. (2022) | oral production task | quasi | n/a | no | no | yes | yes (ICC = .89) | no | yes | no |
| Papanagnou et al. (2016) | IV placement (cannulation) | randomized | no | no | n/a | yes | not reported | no | yes | no |
| Rassaei (2018) | vocabulary test--cued production | randomized | no | no | n/a | yes | test, retest is .76, inter-rater is .96 | no | yes | no |
| Rassaei (2018) | vocabulary test--multiple choice | randomized | no | no | n/a | yes | test retest is .77 | no | yes | no |
| Rassaei (2020) | oral production task | randomized | no | no | n/a | yes | test, retest is .73, inter-rater agreement is .88 | no | yes | no |
| Rassaei (2020) | writing task | randomized | no | no | n/a | yes | test-retest is .75, interrater agreement is .91 | no | yes | no |
| Riding & Douglas (1993) | short recall | randomized | no | no | n/a | yes | not reported | no | yes | no |
| Riding & Douglas (1993) | explanation question | randomized | no | no | n/a | yes | not reported | no | yes | no |
| Riding & Douglas (1993) | problem solving question | randomized | no | no | n/a | yes | not reported | no | yes | no |
| Riding & Douglas (1993) | labeling questions | randomized | no | no | n/a | yes | not reported | no | yes | no |
| Rogowsky et al. (2015) | multiple choice questions | randomized | no | no | n/a | yes | test-retest is .86 | no | yes | no |
| Rogowsky et al. (2020) | Reading test | within (counterbalanced) | no | no | n/a | yes | alternate form reliability is .86, internal consistency is .92-.93 | no | yes | no |
| Tadayonifar et al. (2021) | Vocabulary test | within (counterbalanced) | no | no | n/a | yes | Cronbach’s alpha = .82 | no | yes | no |

**Table B2**

Study quality ratings

| **Study** | **Rating** |
| --- | --- |
| Aslaksen & Lorås (2019), journal article | Does not meet WWC standards |
| Burns (n.d.), unpublished manuscript | Does not meet WWC standards |
| Chen (2020), journal article | Does not meet WWC standards |
| Chen & Sun (2012), journal article | Does not meet WWC standards |
| Chui et al. (2021), journal article | Does not meet WWC standards |
| Cuevas & Dawson (2018), journal article | Meets WWC standards without reservations |
| Ge (2021), journal article | Does not meet WWC standards |
| Hazra et al. (2013), conference proceedings | Does not meet WWC standards |
| Kam et al. (2020) | Does not meet WWC standards |
| Kassaian (2007), journal article | Does not meet WWC standards |
| Lehmann & Seufert (2020), journal article | Does not meet WWC standards |
| Moser & Zumbach (2018), journal article | Does not meet WWC standards |
| Moussa-Inaty et al. (2019), journal article | Does not meet WWC standards |
| Mujtaba et al. (2022), journal article | Does not meet WWC standards |
| Papanagnou et al. (2016), journal article | Does not meet WWC standards |
| Rassaei (2018), journal article | Meets WWC standards without reservations |
| Rassaei (2020), journal article | Meets WWC standards without reservations |
| Riding & Douglas (1993), journal article | Does not meet WWC standards |
| Rogowsky et al. (2015), journal article | Meets WWC standards without reservations |
| Rogowsky et al. (2020), journal article | Meets WWC standards without reservations |
| Tadayonifar et al. (2021), journal article | Meets WWC standards without reservations |
